# Rapid and extensive karyotype diversification in haploid clinical *Candida auris* isolates

**DOI:** 10.1007/s00294-019-00976-w

**Published:** 2019-04-24

**Authors:** Gustavo Bravo Ruiz, Zoe K. Ross, Eilidh Holmes, Silke Schelenz, Neil A. R. Gow, Alexander Lorenz

**Affiliations:** 1 Institute of Medical Sciences (IMS), University of Aberdeen, Foresterhill, Aberdeen, AB25 2ZD UK; 2 MRC Centre for Medical Mycology, University of Aberdeen, Aberdeen, UK; 3 Department of Microbiology, Royal Brompton Hospital, London, UK; 4 Present Address: School of Biosciences, University of Exeter, Exeter, UK

**Keywords:** *Candida auris*, Chromosome number, Chromosome size, Genome size, Karyotype evolution

## Abstract

*Candida auris* is a newly emerged pathogenic microbe, having been identified as a medically relevant fungus as recently as 2009. It is one of the most drug-resistant yeast species known to date and its emergence and population structure are unusual. Because of its recent emergence, we are largely ignorant about fundamental aspects of its general biology, life cycle, and population dynamics. Here, we report the karyotype variability of 26 *C. auris* strains representing the four main clades. We demonstrate that all strains are haploid and have a highly plastic karyotype containing five to seven chromosomes, which can undergo marked alterations within a short time frame when the fungus is put under genotoxic, heat, or osmotic stress. No simple correlation was found between karyotype pattern, drug resistance, and clade affiliation indicating that karyotype heterogeneity is rapidly evolving. As with other *Candida* species, these marked karyotype differences between isolates are likely to have an important impact on pathogenic traits of *C. auris*.

**Electronic supplementary material:**

The online version of this article (10.1007/s00294-019-00976-w) contains supplementary material, which is available to authorized users.

## Introduction

A current, major concern in medical mycology is the emergence of the multidrug-resistant pathogen *Candida auris.* This species was named according to its first identification as an isolate from the ear canal of a Japanese patient in 2009 (Satoh et al. 2009). Since then, it has rapidly become a major healthcare threat with hospital outbreaks occurring worldwide (Chowdhary et al. 2016; Jeffery-Smith et al. 2018). Most *C. auris* isolates show high levels of resistance to antifungal drugs, including azoles, echinocandins, 5-flucytosine, and polyenes (amphotericin B) (Chakrabarti et al. 2014; Lockhart et al. 2017). *C. auris* is also difficult to eradicate from hospital intensive care wards and as a skin colonizer it can apparently be transmitted from patient to patient (Jeffery-Smith et al. 2018).

Whole-genome sequencing (WGS) of *C. auris* isolates has indicated that there are at least four distinct geographical clades of this species; East Asia (Japan, Korea), South Asia (India, Pakistan), South Africa, and South America (Venezuela) (Lockhart et al. 2017). Clades differ by tens of thousands of single-nucleotide polymorphisms (SNPs) from each other; however, within each clade, isolates are almost indistinguishable from each other on a DNA sequence level (Chakrabarti et al. 2014; Lockhart et al. 2017; Rhodes et al. 2018). This suggests that the *C. auris* population structure is characterized by distinct and highly variable clades that are distributed worldwide and almost non-variable clonal expansions of a single genotype within individual outbreaks (Jeffery-Smith et al. 2018). The origin(s) of the strong variability between and the minor variability within clades are currently unknown.

Polyploidy, aneuploidy, and gross chromosome rearrangements have been recognized as drivers of genetic diversity in pathogenic and non-pathogenic fungi for some time (Zolan 1995; Fierro and Martín 1999; Bennett et al. 2014; Wertheimer et al. 2016; Monerawela and Bond 2017; Harari et al. 2018a). In pathogenic yeasts, such as *C. albicans*, mechanisms for ploidy shifts and chromosome rearrangements have been described, and their importance for adaptation to environmental stresses and host niches, as well as for developing resistance to antifungal drugs has been identified (Selmecki et al. 2006, 2009; Wertheimer et al. 2016).

Here, we characterize a set of 26 clinical isolates of the newly emerging human pathogenic fungus *C. auris* to understand whether its genome has undergone structural alterations potentially underlying adaptation events. This strain collection covers all four geographical clades, different levels of drug resistance, and various sources of isolation (Table S1). All isolates were shown to be haploid, and we observed substantial karyotypical variability between *C. auris* strains, even between isolates belonging to the same clade. Importantly, genetic diversity on a DNA sequence level within a clade had been reported to be minimal (Chakrabarti et al. 2014; Lockhart et al. 2017; Rhodes et al. 2018). We also tested whether under heat, osmotic, or DNA replication stress karyotype changes are induced in *C. auris*, similar to other fungi (Todd et al. 2017). The frequency of changes is higher in stress conditions, but minor alterations could also appear when *C. auris* was grown in standard laboratory conditions. We observed that in some cases these changes are associated with fitness benefits. However, other karyotype modifications seem to be stochastic and would not confer an advantage, as previously reported for other fungi (Rustchenko et al. 1997; Janbon et al. 1999).

## Materials and methods

### Yeast strains and culture

*Candida auris* and other yeast strains used in this study are listed in Table S1. *Candida albicans* SC5314, and *Saccharomyces cerevisiae* BY4741 and BY4743 were used as control organisms. Yeast cells were grown at 30 °C on YPD plates (1% yeast extract, 2% mycological peptone, 2% glucose, 2% agar; Oxoid, Basingstoke, UK) or shaking at 200 rpm in YPD broth (same as plates, but without agar).

### Flow cytometry

Processing yeast samples for flow cytometry was performed largely as previously described (Fortuna et al. 2001). Briefly, stationary-phase yeast cells were inoculated into fresh YPD broth and incubated while shaking (200 rpm) at 30 °C for 3 h. Cells were harvested by centrifugation (1000 × *g*, 2 min), re-suspended at a concentration of 1 × 10^7^ cells/ml in ice-cold demineralized water, and fixed overnight by adding 100% ethanol to a final concentration of 70% ethanol. Cells were then harvested by centrifugation (1000 × *g*, 2 min) and re-suspended in 50 mM sodium citrate (pH 7.5). After RNase A (250 μg per 1 × 10^7^ cells) and proteinase K (1000 μg per 1 × 10^7^ cells) treatment, cells were transferred to 12 × 75 mm round-bottom tubes. After adding Triton-X 100 (Sigma-Aldrich) to a final concentration of 0.25%, and SYBR Green I (1:500; Sigma-Aldrich, St. Louis, MI, USA) as a DNA stain, samples were incubated at 4 °C overnight. Before flow cytometry, samples were sonicated (three pulses at 30–60 W for 1–2 s; Sonicator Ultrasonic Processor S-4000, Misonix, Farmingdale, NY, USA). Flow cytometry was performed on a BD LSR II flow cytometer (BD Biosciences, San Jose, CA, USA) using an excitation wavelength of 488 nm, SYBR Green I fluorescence was detected with a 530/30 band pass filter. 10,0000 events, gated for singlets, were recorded for every sample. Data were analyzed using FlowJo 10.2 software (FlowJo LLC, Ashland, OR, USA).

### Pulsed-field gel electrophoresis (PFGE)

Chromosomal DNA of *C. auris* strains was embedded in agarose plugs using the CHEF Genomic DNA Plug Kit (Bio-Rad Laboratories Ltd., Hercules, CA, USA) following the instructions of the manufacturer. For some strains, the cell wall digestion reaction was supplemented with Lallzyme MMX (end concentration 100 mg/ml; Lallemand Inc., Quebec, Canada). Pulsed-field gel electrophoresis (PFGE) was performed on a CHEF Mapper XA System (Bio-Rad). As a standard programme *C. auris* DNA was run for 48 h at 14 °C in 1 × TAE (40 mM Tris, 20 mM acetic acid, 1 mM EDTA; pH 8.0) and 0.8% Megabase agarose (Bio-Rad) at 3.0 V/cm applied at a 106° angle and a switch time of 500 s; every strain and isolate were run at least twice under these conditions (images are available at https://dx.doi.org/10.6084/m9.figshare.7881167). To get a better separation of smaller chromosomes, DNA from selected *C. auris* strains was run for 48 h at 14 °C in 1 × TAE and 0.8% Pulsed-Field Certified agarose (Bio-Rad) at 4.0 V/cm applied at a 120° angle, initial and final switch times of 120 s and 240 s using linear ramping. Gels were stained with SYBR Green I (Sigma-Aldrich) diluted 1:10,000 in 1 × TAE for at least 1 h and documented by photography under UV illumination on a Gel Doc EQ system controlled by Quantity One software (version 4.6.6) (Bio-Rad).

### Southern blot analysis

Chromosome-sized DNA bands from PFGE gels were transferred to Zeta-Probe GT membranes (Bio-Rad) by alkaline Southern blotting following previously described principles (Sambrook and Russell 2000). Gels were soaked in depurinating solution (0.25 M HCl) for ~ 25 min and, after that, in denaturing solution (1.5 M NaCl, 0.5 M NaOH) for ~ 30 min. Using capillary transfer in denaturing solution for 24 h chromosomal DNA was immobilized on the membranes. After transfer, membranes were steeped in neutralization buffer (0.5 M Tris, pH 7.0) for 5 min, washed briefly in 2 × SSC (saline–sodium citrate; 300 mM NaCl, 30 mM Na_3_C_6_H_5_O_7_, pH 7.0) and dried at room temperature.

A fragment of 836 bp was amplified by polymerase chain reaction (PCR) from the 25S rRNA region of *C. auris* strain UACa11 genomic DNA using oligonucleotides oUA367 (5′-GGCAAAACAAAGGCCGCGC-3′) and oUA368 (5′-AGTAGCTGGTTCCTGCCGAAG-3′). This fragment was used as template for a labeling PCR incorporating digoxigenin-11-dUTP with nested primers oUA371 (5′-CCAATTCCAGGGTCACAGGCT-3′) and oUA372 (5′-CCTCAGGATAGCAGAAGCTCGT-3′) to give an rDNA probe of 759 bp (DIG DNA Labeling Mix; Roche Molecular Systems Inc., Pleasanton, CA, USA). All PCR reactions were carried out using GoTaq^®^ G2 Flexi DNA Polymerase (Promega Corp., Madison, WI, USA). Oligonucleotides were supplied by Sigma-Aldrich Co. (St. Louis, MO, USA).

Membranes were hybridized with the digoxigenin-11-dUTP labeled rDNA probe using DIG DNA Labeling Kit (Roche Molecular Systems Inc.), and then incubated with α-digoxigenin antibody (Roche Molecular Systems Inc.) conjugated to alkaline phosphatase. Alkaline phosphatase bound to digoxigenin-11-dUTP labeled bands was then detected on a FUSION SL Chemiluminescence Imaging System (Vilber Lourmat, Marne-la-Vallée, France) using CPD-Star chemi-luminescent substrate (Roche Molecular Systems Inc.).

### Microevolution assay

A microevolution assay was carried out using selected strains from each clade (UACa11, UACa18, UACa20, and UACa22) to test whether karyotype variation can be induced by particular growth conditions: strains were separately passaged five times through YPD broth at 30 °C (control), YPD broth at 42 °C (heat stress), synthetic-defined liquid medium containing 2% sorbose (SSD) (6.7 g/l yeast nitrogen base with amino acids, Sigma-Aldrich) at 30 °C (osmotic stress), and YPD broth containing 100 mM hydroxyurea (HU; Formedium, Norfolk UK) at 30 °C (DNA replication stress). All passages were performed following the same experimental strategy (Fig. S1). Parental strains, from glycerol stock, were grown on YPD plates overnight at 30 °C;  ~ 10^7^ cells from this plate were used to initiate the different passages (Fig. S1). A passage consisted of (1) growing cells in liquid culture overnight under treatment conditions, (2) plating 100–200 cells from that liquid culture on appropriate solid medium until single colonies were visible (2–3 days, except on 2% sorbose where incubation took 1 week), (3) suspending the five largest single colonies in 1 ml of sterile water, determining cell concentration, and making four 1:10 serial dilutions, from 10^2^ to 10^5^ cells, to test resulting isolates in spot assays under the same conditions, (4) selecting the three fastest-growing isolates for long-term storage (40% glycerol at − 70 °C), and (5) inoculating the fastest-growing isolate at ~ 10^7^ cells in a fresh overnight liquid culture starting the next passage (Fig. S1).

Isolates from the first and the fifth passage of each strain and condition were subjected to PFGE analysis (see above), and spot assays under the same conditions used for each passage (except for DNA replication stress where isolates were also tested on plates containing a higher concentration of 200 mM HU); the parental strain was always included for comparison. Isolates from the fifth passage in sorbose were also tested on YPD plates containing 4 and 16 μg/ml caspofungin (CSP) at 30 °C. For the DNA preparation for PFGE analysis and the spot assays, isolates and parental strains were revived from long-term storage on YPD agar (overnight at 30 °C) and then grown in YPD broth overnight at 30 °C. Appropriate plates were grown for 1–6 days depending on conditions and temperature used for each passage.

Cell concentrations were determined by measuring optical density of the culture at a wavelength of 600 nm (OD_600_) on an Ultraspec 2000 (Pharmacia Biotech, Sweden) spectrometer. Previous calibration defined a *C. auris* culture of OD_600_ = 1 to contain 3 × 10^7^ cells/ml.

## Results

### *Candida auris* is a haploid fungus

Polyploidy and complex aneuploidy play a major role in the capability of fungal pathogens to adapt to various stresses and to the changing condition within host niches (Bennett et al. 2014; Wertheimer et al. 2016). These overall genome shifts in chromosome number have been characterized as drivers of increased genetic diversity in *Cryptococcus neoformans*, *Candida albicans*, and also in *Candida lusitaniae*—a close relative of *C. auris* (Forche et al. 2008; Reedy et al. 2009; Ni et al. 2013; Hirakawa et al. 2017).

**Fig. 1:**
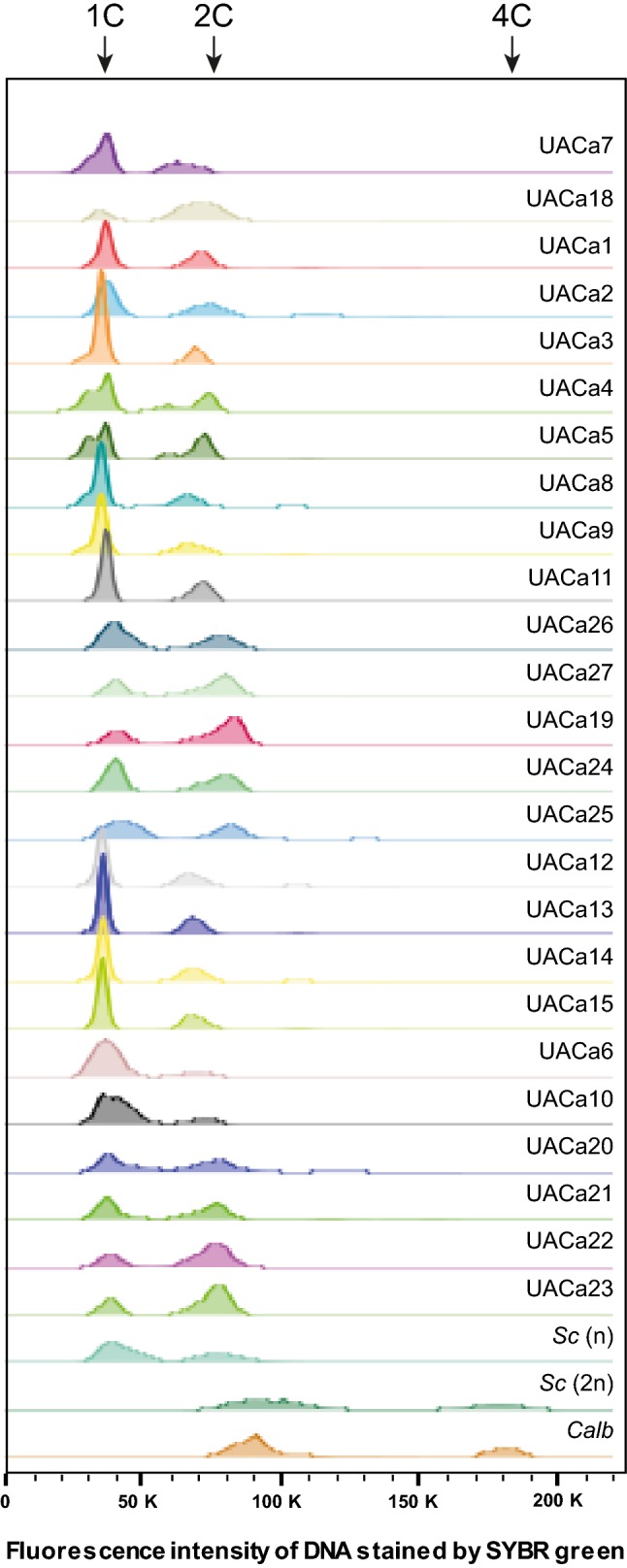
Cell cycle profiles of clinical *Candida auris* isolates. Histogram showing cell cycle profiles obtained by flow cytometry after staining DNA with SYBR green of 25 *C. auris* strains (Table S1). Diploid *Candida albicans* (*Calb*) strain SC5314, haploid (n, BY4741) and diploid (2n, BY4743) strains of *Saccharomyces cerevisiae* (*Sc*) were included as controls. *C. auris* has the same cell cycle profile as haploid *S. cerevisiae*. *C. auris* strains are grouped according to their taxonomical position within the four geographical clades: E. Asia (UACa7 & UACa18), S. Asia-India (UACa1-5, UACa8, UACa9, UACa11, UACa26-27), S. Asia-Pakistan (UACa19, UACa24-25), strains from the Royal Brompton hospital outbreak (UACa12-15), S. Africa (UACa6, UACa10, UACa20-21), and S. America (UACa22-23). Approximate position of haploid G1 (1C), haploid G2 and diploid G1 (2C), and diploid G2 (4C) peaks are indicated at the top

Therefore, we were interested in determining the ploidy of clinical *C. auris* isolates to understand whether chromosome number variations and whole-genome duplication potentially are adaptive strategies employed by *C. auris*. In total 25 *C. auris* strains covering all four geographical clades, comprising antifungal-sensitive, -resistant, and -multiresistant isolates from various sources of infection, as well as strains from a single outbreak (Royal Brompton hospital, London, UK) (Table S1) were tested by flow cytometry (Chakrabarti et al. 2014; Sharma et al. 2015; Schelenz et al. 2016; Lockhart et al. 2017). Haploid and diploid *Saccharomyces cerevisiae* strains and the diploid *C. albicans* laboratory strain SC5314 were used as references. Estimates from whole-genome sequencing suggested that *C. auris* has a similar genome size as *S. cerevisiae* at approx. 12 Mbp (Goffeau et al. 1996; Lockhart et al. 2017). As expected, *C. albicans* SC5314 had a similar cell cycle profile as a diploid *S. cerevisiae* strain (Fig. 1). In contrast, the genome size of all 25 *C. auris* strains was found to be consistent with containing a haploid chromosome complement (Fig. 1), although the resolution of flow cytometry probably does not allow us to unequivocally exclude the occasional disomy of one of the smaller chromosomes. However, in none of the 25 strains, disomies were apparent in the karyotype analysis (see below).

This result indicates, that major ploidy changes do not appear to be a mechanism *C. auris* employs to adapt to the environmental challenges tested here. If it does, this must be only temporarily with diploids, polyploids, or aneuploids returning quickly to a haploid stage after these challenges are removed.

### *Candida auris* clinical isolates have a plastic karyotype

**Fig. 2:**
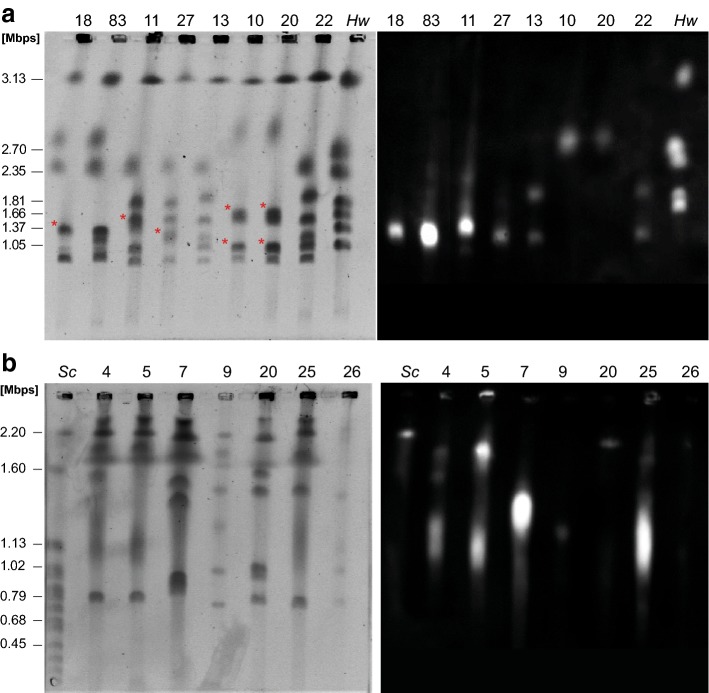
Representative PFGE karyotypes of clinical *Candida auris* isolates. PFGE karyotypes (left panels, inverted image) and associated Southern blots using a rDNA probe to detect rRNA gene clusters (right panels) of the indicated strains representing examples of E. Asian (UACa7, UACa18, UACa83), S. Asian (UACa4, UACa5, UACa9, UACa11, UACa25, UACa26, UACa27), S. African (UACa10, UACa20), and S. American (UACa22) clades, as well as one isolate from the outbreak at the Royal Brompton Hospital, London, UK (UACa13) (Table S1). **a** Gel run at standard conditions (0.8% Megabase agarose, 1 × TAE, 48 h, 14 °C, 3.0 V/cm, 106°, switch time 500 s), *Hansenula wingei* (*Hw*) CHEF DNA size marker (Bio-Rad) serving as standard (size of chromosomal bands in Mbp indicated on the left). Single bands considered as two chromosomes are indicated by red asterisks (*). **b** Gel run at conditions to resolve smaller chromosomes (0.8% Pulsed Field Certified agarose, 1 × TAE, 48 h, 14 °C, 4.0 V/cm, 120°, switch times: linear ramping 120–240 s), *Saccharomyces cerevisiae* (*Sc*) CHEF DNA size marker (Bio-Rad) serving as standard (size of some chromosomal bands in Mbp indicated on the left)

**Fig. 3:**
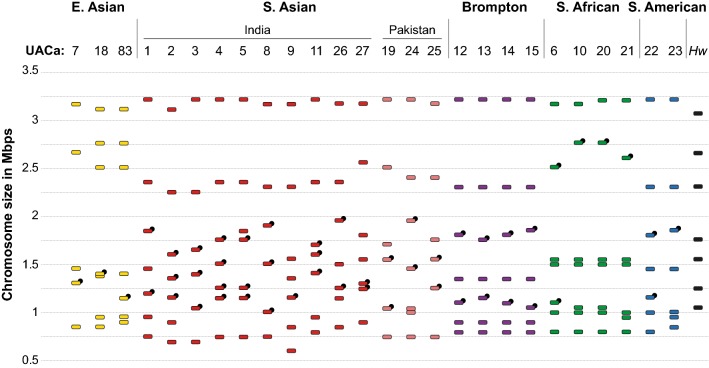
Schematic of karyotypes of 26 clinical *Candida auris* isolates. Strains are grouped according to their taxonomical position within the four geographical clades. Chromosomes are represented as differently coloured rectangles to indicate affiliation to a particular clade: E. Asia (yellow), S. Asia (India, red; Pakistan, light red), S. Africa (green), and S. America (blue); the four strains isolated from the outbreak at the Royal Brompton Hospital, London (Brompton) are indicated as a separated group (purple). Black rectangles indicate the chromosomes of *Hansenula wingei* (*Hw*). Chromosome sizes were measured using the *Hansenula wingei* (*Hw*) CHEF DNA size marker (Bio-Rad) and strain UACa11 as standards, both of which were run alongside the samples in each gel. Chromosomes harboring rRNA gene clusters are indicated by a black circle (●). See https://dx.doi.org/10.6084/m9.figshare.7881167 for gel images

Since all the *C. auris* isolates tested turned out to be haploid (or near haploid), we wondered whether its genetic diversity might be generated by gross chromosome rearrangements. To test this we utilized PFGE to separate *C. auris* chromosomes (Fig. 2). We characterized the same 25 strains covering all four geographical clades and the single hospital outbreak as above, as well as an additional East Asian isolate, UACa83 (the type-strain of *C. auris*, CBS10913T) (Table S1) (Satoh et al. 2009; Chakrabarti et al. 2014; Sharma et al. 2015; Schelenz et al. 2016; Lockhart et al. 2017). 11 of these strains have previously been whole-genome sequenced (Sharma et al. 2015; Lockhart et al. 2017), and two of these (UACa20, and UACa24) had their genomes assembled into seven chromosome-size contigs (Muñoz et al. 2018). *C. auris* isolates show chromosome numbers from 5 to 7, ranging from ~ 0.7 Mbp to ~ 3.25 Mbp in size (Figs. 2, 3); chromosomes will be referred to by their size. Additionally, we probed the karyotypes for the location of the repetitive rRNA gene clusters (rDNA) by Southern blotting (Figs. 2, 3). It should be noted that chromosomal bands of similar size are difficult to separate using the PFGE system; however, we have considered the presence of two chromosomes when the band intensity is clearly higher, e.g., chromosomal band around 1 Mbp in UACa20 likely or chromosomal band around 1.35 Mbp in UACa18 possibly each contain two chromosomes.

The E. Asian strains of this study apparently have five to seven chromosomes one of which carries rRNA gene repeats. Intriguingly, UACa18 (B11220 obtained from the CDC, Atlanta, GA, USA) and UACa83 (CBS10913T obtained from the Westerdijk Fungal Biodiversity Institute, Utrecht, The Netherlands) originate from the same type material (Satoh et al. 2009), but do show slight karyotypical differences in the smaller chromosomes (Fig. 3). The S. African and S. American isolates we studied all had seven chromosomes and with the exceptions of UACa6 (S. Africa) and UACa22 (S. America) only one of the chromosomes was rDNA-bearing (Fig. 3). In contrast, chromosome numbers in S. Asian isolates ranged from six to seven, and, except for UACa9, all S. Asian strains had at least two chromosomes carrying rRNA gene repeats (Fig. 3). The range of chromosome numbers and of chromosome size distributions in S. Asian isolates may reflect the comparatively large intra-clade genetic diversity of this strain cluster (Lockhart et al. 2017), but we cannot exclude this to be an issue of having only a small sample set available for non-S. Asian isolates. The four strains from the Royal Brompton hospital outbreak (UACa12–UACa15) had similar karyotypes to each other with seven detectable chromosomal bands. In these strains the two chromosomes bearing rDNA repeats displayed subtle size differences between these particular set of strains (Fig. 3). Interestingly, the four strains from the Royal Brompton hospital outbreak appeared similar to S. Asian strains, which they are also most closely related to according to whole-genome sequence analysis (Rhodes et al. 2018).

Adding chromosome-size estimates from PFGE points towards a range of genome sizes between ~ 10 Mbp and ~ 13 Mbp which is a reasonable fit to the 12.5 Mbp suggested by whole-genome sequencing (Lockhart et al. 2017), and conforms with our flow cytometry results (Fig. 1). Complete assembly of whole-genome sequences into chromosome-sized contigs to create physical maps of *C. auris* genomes will allow full appreciation of the genome structure of this fungus. Indeed, a recent study reported seven contigs for two *C. auris* isolates, in our PFGE analysis the corresponding strains UACa20 (B11221) and UACa24 (B8441) also display seven chromosomal bands similar in size to the reported contigs (Muñoz et al. 2018).

Electrophoretic karyotyping revealed that *C. auris* isolates differed considerably in chromosome numbers and sizes, both within and between geographical clades. This plasticity was somewhat unexpected considering the genetic uniformity of *C. auris* on a DNA sequence level within geographical clades and within hospital outbreaks, and indicates that gross chromosome rearrangements might be a mechanism *C. auris* employs to generate genetic diversity during adaptation to environmental challenges (Sharma et al. 2016; Lockhart et al. 2017; Rhodes et al. 2018).

### *C. auris* undergoes karyotype rearrangements in stress conditions

To get insight about *C. auris* fitness and its relation to the karyotype variation observed in different clinical isolates, four strains (UACa11, UACa18, UACa20, and UACa22), one from each clade (Table S1), were selected to undergo a microevolution experiment. Strains were grown under four different conditions through five passages each (see “Materials and Methods”, Fig. S1): standard YPD broth at 30 °C (control); heat stress at 42 °C; osmotic stress using 2% sorbose, which mimics the effect of echinocandin-type antifungals; and DNA replication stress using hydroxyurea (HU), an inhibitor of the enzyme ribonucleotide reductase which depletes nucleotide pools (Koç et al. 2004; Yang et al. 2013). These conditions have been described previously as factors inducing genome instability in fungi. Introduction of heat stress has been shown to induce ploidy variation in *C. albicans* and other fungal species (Anderson et al. 2015). In *C. albicans*, treatment with 2% sorbose is a classic example for inducing changes in the karyotype, which offer a phenotypic advantage in this stress (Rustchenko et al. 1994; Janbon et al. 1999; Kabir et al. 2005). Replication stress, for example by treatment with HU, has been associated with genome instability in a wide range of organisms, including yeast and human cells (Arlt et al. 2011; Maxwell 2016).

**Fig. 4:**
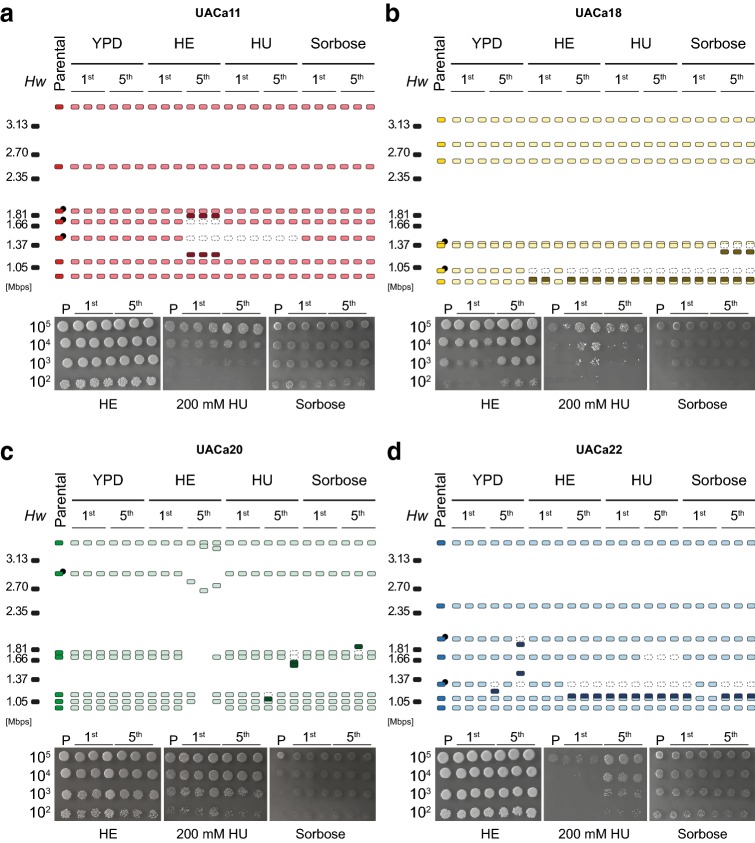
Karyotype variation during microevolution of *Candida auris* isolates. Schematic representation of karyotypes (top panels) and spot assays (bottom panels) of four *C. auris* isolates covering all four established clades (**a**) UACa11 (S. Asia), (**b**) UACa18 (E. Asia), (**c**) UACa20 (S. Africa), and (**d**) UACa22 (S. America)—obtained after the first and fifth passages in four different growth conditions: YPD at 30 °C; YPD at 42 °C, heat stress (HE); YPD containing 100 mM hydroxyurea at 30 °C (HU); and 2% sorbose in synthetic defined medium at 30 °C (sorbose). Chromosomes are represented as coloured rectangles for different strains or black rectangle for *Hansenula wingei* (*Hw*) CHEF DNA size marker (Bio-Rad) used as a chromosome size standard (numbers represent size in Mbp). Chromosomal bands that seemingly disappear in comparison to parental strains are represented as empty rectangles with dotted lines. Darker rectangles represent new chromosomal bands appearing in comparison to parental strains. In strain UACa20 under heat stress the karyotype changes observed indicate massive chromosome rearrangements. Chromosomes harboring rRNA gene clusters are indicated by a black circle (●) only in parental strains. Spot assays show the parental strain (P) and derived isolates for comparison, grown in the same conditions used for the respective passages, except for HU for which a higher concentration (200 mM) than during the passages (100 mM) is used. Serial dilutions contain 10^5^, 10^4^, 10^3^, and 10^2^ cells. Isolates were grown for 1 day in heat stress (42 °C), 3 days in HU (due to slow growth, UACa18 and its derivatives were grown for 6 days), or 3 days in 2% sorbose. See https://dx.doi.org/10.6084/m9.figshare.7881167 for gel images

In our microevolution study, karyotype modifications including appearance, disappearance, or size changes of different chromosomal bands were observed in all tested strains. However, the frequency and type of modification were different depending on the strain and condition used (Fig. 4).

The S. Asian strain UACa11 has shown chromosome modifications under heat and DNA replication (HU) stress conditions, always related to chromosomes carrying rDNA repeats (Fig. 4a). In HU, the 1.35 Mbp chromosomal band seemingly disappears after only one passage. In heat stress, the 1.35 Mbp and 1.6 Mbp chromosomal bands disappeared, and two additional ones appeared (around 1.1 Mbp and 1.7 Mbp), most likely due to loss and gain of DNA from the corresponding original chromosomes.

The E. Asian isolate UACa18 showed a tendency to lose the 0.95 Mbp chromosomal band in all stress conditions, but not when growing in YPD at 30 °C (control), most likely changing in size to a slightly smaller band of around 0.9 Mbp (Fig. 4b). When treated with sorbose, a new chromosome of around 1.3 Mbp appeared after five passages in UACa18, likely because of the reduction in size of one of the two 1.35 Mbp chromosomes.

Looking at the S. African strain UACa20, only minor changes were observed after five passages in sorbose and HU (Fig. 4c). Specifically, one alteration was observed in sorbose where one of the isolates seemed to gain DNA in the 1.65 Mbp chromosome, in HU where the 1.05 Mbp chromosome decreased in size in one isolate, and two other chromosomes (1.6 and 1.65 Mbp) seemingly reduced size in another isolate. However, under heat stress conditions drastic changes were observed, the number of chromosomes reduced from seven to six, four, or three bands in three different isolates. Importantly, flow cytometry demonstrated that these substantial karyotype changes did not cause any conspicuous alterations of the ploidy state (Fig. S2).

Finally, the S. American strain UACa22 was the most plastic, showing karyotype changes even when grown in control conditions (YPD, 30 °C), in this case chromosomes carrying the rDNA tend to change size, losing or gaining DNA (Fig. 4d). In almost all conditions the 1.2 Mbp chromosome tends to reduce in size. Besides, a second chromosomal band (1.6 Mb) was seemingly lost in the presence of HU after five passages.

Despite the karyotype alterations observed, a moderate improvement in growth was detected only in a few cases (Fig. 4): isolates from UACa18 microevolved in 2% sorbose from the first passage, two isolates from UACa18 after one passage in 100 mM HU; and isolates from UACa22 after five passages of 100 mM HU when tested at a higher concentration of 200 mM HU. These improvements in growth could be correlated with changes in the karyotype in some cases (see above). However, this clearly is not probable for all the cases, e.g., isolates of strain UACa20 that grow better in sorbose, but did not show any difference in karyotype in comparison to the parental strain, and modifications in karyotype that did not obviously offer any improved fitness in the condition tested. Interestingly, the massive karyotype variation observed in microevolved UACa20 isolates obtained under heat stress did not show an apparent difference growing at 42 °C compared to the parental strain. Isolates from the strain UACa11 did not show improvement in growth under any conditions. Because osmotic stress in 2% sorbose mimics the effect of echinocandins, we tested whether growth of isolates obtained from the fifth passage in 2% sorbose displayed improved growth in the presence of caspofungin (CSP) (Fig. S3). Isolates derived from the CSP-sensitive UACa18 strain had undergone karyotype alterations and showed a clear improvement in growth both on 2% sorbose and in the presence of CSP (Figs. 4b, S3). However, this was different for the other microevolved isolates: derivatives of UACa11 showed slow growth already on 4 μg/ml CSP in comparison to the parental strain (Fig. S3), without any distinguishable growth defect on 2% sorbose (Fig. 4a). Isolates from strains UACa20 and UACa22 were highly resistant to CSP similar to the parental strains, and original and derived isolates showed little differences in growth on 2% sorbose (Figs. 4c, d, S3).

## Discussion

Here, we show that the genome of *C. auris* undergoes substantial karyotypic reorganization under stress conditions (Fig. 4), similar to other fungi (Ormerod and Fraser 2013; Ahmad et al. 2014; Wertheimer et al. 2016). Karyotype variation between different strains of the same species is common in diploid *C. albicans* (Chu et al. 1993; Magee and Magee 1997) and haploid *Schizosaccharomyces pombe* (Brown et al. 2011; Jeffares et al. 2017), and it has been proposed as a quick solution for adaptation to environmental changes. This variation of genome organization can take on different expressions, e.g., ploidy variation, chromosome copy number variation, or gain and loss of supernumerary chromosomes (Covert 1998; Tang and Amon 2013; Bennett et al. 2014; Todd et al. 2017; Zhang and Ma 2017; Harari et al. 2018b). High levels of genetic diversity can be introduced into a population by changes in ploidy. In yeast most of these changes produce a mixture of aneuploid populations offering a rapid solution for stress adaptation, this has been suggested to be the norm in fungi (Bennett et al. 2014). These changes in genome structure might be introduced by various mechanisms usually related to chromosome segregation mistakes during mitosis, meiosis, or during parasexual reproduction. Once ploidy changes arise in *S. cerevisiae*, rates of chromosome loss, genetic mutation, and microsatellite instability increase, usually leading to proliferative disadvantages (Torres et al. 2007, 2010; Forche et al. 2008; Sheltzer and Et 2011). Having said that, aneuploid and polyploid isolates of *S. cerevisiae* exposed to a range of stress conditions can display a growth advantage (Pavelka et al. 2010; Harari et al. 2018b); however, aneuploidy is often lost, when the stress is eliminated (Janbon et al. 1998). *C. auris* is haploid (Fig. 1), and so far, neither polyploid states nor sexual reproduction have been described. Therefore, we hypothesize that *C. auris* is likely not capable of generating genome diversity via aneuploidy or polyploidy. Further studies to elucidate the life cycle of *C. auris* will be necessary to shed light on this issue.

As a haploid species, the variation observed in *C. auris* would most likely be due to gross chromosome rearrangements, and/or possibly copy number variation (CNV) events of chromosomal sections. CNVs are a frequent reason for changes in the genome organization of haploid fungi (Zhang et al. 2013; Ahmad et al. 2014). CNVs of yet-to-be-identified genomic regions, potentially repetitive, could explain the changes in size observed in the karyotype of *C. auris*, e.g., the 0.95 Mbp chromosomal band in UACa18, or 1.2 Mbp chromosome in UACa22 (Fig. 4). In general, repetitive regions are known as a principal reason for CNVs and gross chromosome rearrangements in yeasts, such as transposons (Ty elements and solo LTR elements in *S. cerevisiae*), telomeres, or rDNA (Mieczkowski et al. 2006; Maxwell et al. 2011; Zhang et al. 2013; Kobayashi 2014; Kupiec 2014; Liu et al. 2016). Intriguingly, the appearance of CNVs increases environmental adaptation, for example increased resistance to antifungal azoles in *C. albicans* (Dunham et al. 2002; Selmecki et al. 2006; Measday and Stirling 2016; Hull et al. 2017). One of the best-studied repetitive elements is the highly conserved rDNA locus consisting of a large number of tandemly repeated rRNA genes interspersed with non-coding intergenic regions. In *S. cerevisiae* the rDNA consists of ~ 150 tandem copies of a 9.1-kb sequence, and in *C. albicans* the haploid genome contains a single ~ 12-kb rDNA region located on chromosome R (Keil and Roeder 1984; Jones et al. 2004). The rDNA array is highly recombinogenic undergoing fluctuations in copy number, which in *S. cerevisiae* is related to the loss of global chromosomal stability, especially during senescence (Kobayashi 2008; Pal et al. 2018). Furthermore, in *C. albicans* chromosome R has been described as more unstable than the other chromosomes within the complement, and thus more frequently displaying size changes (Iwaguchi et al. 1992; Rustchenko et al. 1993). Indeed, a set of four strains from the outbreak at the Royal Brompton hospital shows moderate size changes in the chromosomes harboring the rDNA repeats only (Fig. 3). In the first draft genome obtained for *C. auris* strain Ci6684, seven loci containing rRNA gene repeats were described, since this is an incomplete assembly containing 99 scaffolds, the true number of rDNA loci will likely be smaller (Chatterjee et al. 2015). Our survey of PFGE karyotype by Southern blotting using *C. auris* rDNA as a probe indicates that between one and four chromosomes harbor rRNA gene repeats (Fig. 3), which could be the source for some of the rearrangements we observed (Figs. 3, 4). The involvement of repetitive elements other than rDNAs, such as retrotransposon or minisatellites, in chromosome rearrangements and CNVs in *C. auris*, will thus be an interesting aim for future study. As example, the appearance of a novel chromosomal band (1.3 Mb) has been observed in the strain UACa18 after passaging through 2% sorbose (Fig. 4). There are several potential explanations for this observation, among the more likely ones are, (1) that the chromosomal band at 1.37 Mbp contains two chromosomes, (2) that in the microevolved UACa18-derivative the resulting population represents a mixture containing cells with the original rDNA-bearing 1.37 Mbp chromosome and cells with a considerably shorter version of this chromosome, or (3) similar to *C. glabrata*, that the appearance of the novel chromosome originated from segmental duplications in one of the two smaller chromosomes (Fig. 4) (Poláková et al. 2009). These novel chromosomes in *C. glabrata* carry duplicated genes potentially involved in yeast–host interaction and virulence (Poláková et al. 2009).

In our microevolution study, we observed the disappearance of chromosomal bands, which cannot obviously be explained by a change in size, like in strain UACa11 and UACa22 in HU (Fig. 4). In these instances, it is likely that a change in size occurred, and the new size is being masked by another chromosomal band. Due to *C. auris* being haploid, we can exclude a complete loss of a chromosome, as has been described in diploid *C. albicans* and *S. cerevisiae* (Selmecki et al. 2006; Wertheimer et al. 2016; Tutaj et al. 2019).

Strikingly, we observed massive chromosome rearrangements without changes in genome size in the strain UACa20 during our microevolution assay under heat stress reducing the number of chromosomes from seven down to three chromosomes in one isolate (Figs. 4, S2). This demonstrates that drastic modifications of the genome structure do not necessarily impinge on viability in *C. auris*, but might actually provide opportunities for general fitness adaptation. In fungi, two mechanisms have been suggested as a cause for reduction in chromosome numbers, telomere-to-telomere fusions and inactivation of one centromere, or breakage of a chromosome at a centromere and posterior fusion to telomeres of another chromosome (Gordon et al. 2011; Wendland and Walther 2014). Viable strains of *S. cerevisiae* with a genome consisting of only one or two chromosome have been obtained by CRISPR-Cas9-mediated engineering of end-to-end chromosome fusions and centromere deletions, though these strains display a somewhat reduced fitness (Luo et al. 2018; Shao et al. 2018). The isolates obtained from *C. auris* in this study with three, four, or six chromosomes, instead of seven as in the parental strains, did not show any obvious growth defects, and likely are thus fully viable (Fig. 4). Although changes in the karyotype of *C. auris* are not obviously faster than in any other related fungus, our results demonstrate that it is capable of undergoing and maintaining drastic alterations of its genome structure. This could be a source of adaptation to stressful conditions, and could underpin the virulence of this dangerous fungus.

## Electronic supplementary material

Below is the link to the electronic supplementary material.

Supplementary material 1 (PDF 1182 kb)
