# Supplementary material for: Rapid and extensive karyotype diversification in haploid clinical Candida auris isolates
Source: Curr Genet. 2019 Apr 24;65(5):1217–28. doi: 10.1007/s00294-019-00976-w (PMC6744574; doi:10.1007/s00294-019-00976-w)
Supplement: Supplementary file 1 — Supplementary material 1 (PDF 1182 kb) [file 294_2019_976_MOESM1_ESM.pdf]

# Supplementary Materials

## Rapid and extensive karyotype diversification in haploid clinical *Candida auris* isolates

Gustavo Bravo Ruiz[1], Zoe K. Ross[1,2], Eilidh Holmes[1], Silke Schelenz[3], Neil A.R. Gow[1,2,4], and Alexander Lorenz[1,*]

Author affiliations: [1]Institute of Medical Sciences (IMS), University of Aberdeen, Aberdeen, UK

[2]MRC Centre for Medical Mycology, University of Aberdeen, Aberdeen, UK

[3]Department of Microbiology, Royal Brompton Hospital, London, UK

[4]Present address: School of Biosciences, University of Exeter, Exeter, UK

*Correspondence should be addressed to:

Alexander Lorenz

Institute of Medical Sciences (IMS)

University of Aberdeen

Foresterhill

Aberdeen AB25 2ZD

United Kingdom

Phone: +44 1224 437323

E-mail: a.lorenz@abdn.ac.uk

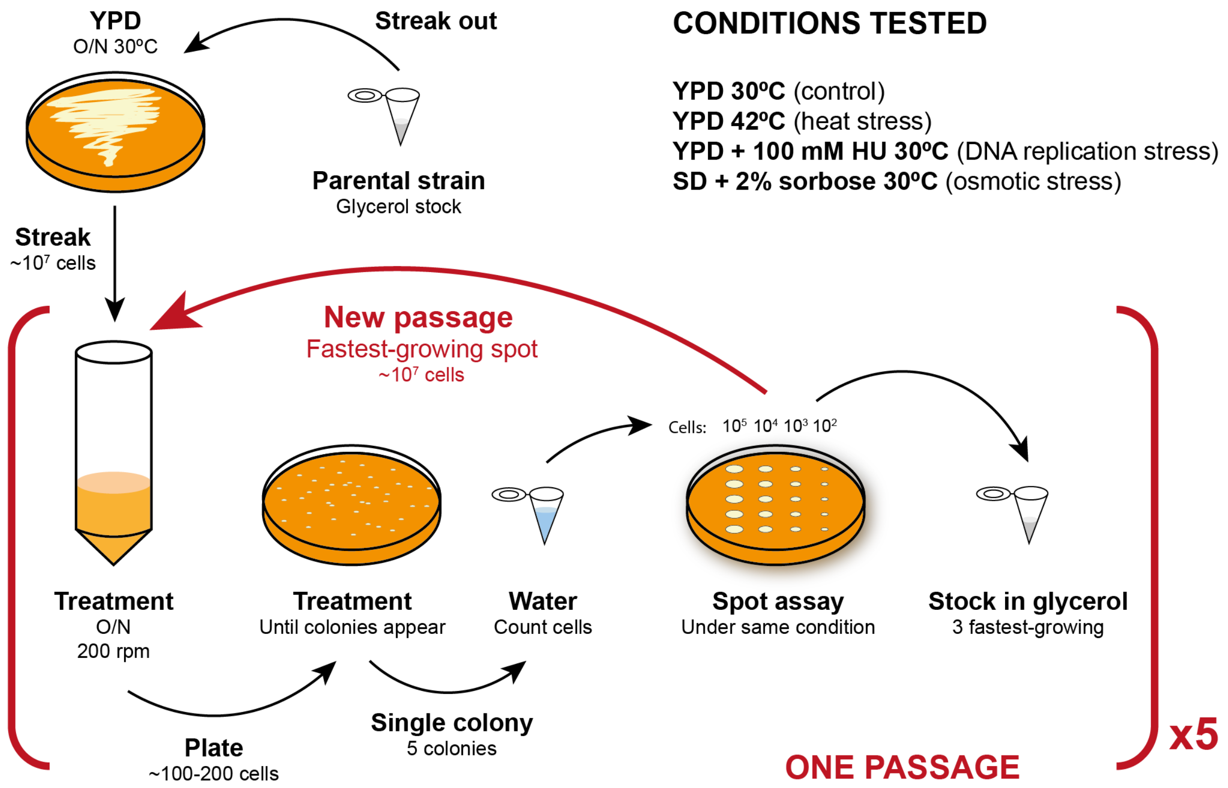


**Figure S1. Microevolution assay.** Schematic representation of the microevolution assay carried out in this study. Strains where separately passaged five times through YPD broth at 30°C (control), YPD broth at 42°C (heat stress), synthetic defined (SD) liquid medium containing 2 % sorbose at 30°C (osmotic stress), and YPD broth containing 100 mM hydroxyurea (HU) at 30°C (DNA replication stress). Parental strains, from glycerol stock, were grown on YPD plates over night at 30°C. ~$10^7$ cells from that plate were inoculated into liquid culture to initiate the different passages. A passage consisted of (1) growing cells in liquid culture overnight under treatment conditions, (2) plating 100-200 cells from that liquid culture on appropriate solid medium (under treatment conditions) until single colonies were visible, (3) selecting the five largest single colonies for spot assays under the treatment conditions, (4) picking the three fastest-growing isolates for long-term storage, and (5) the fastest one to inoculate ~$10^7$ cells in a fresh overnight liquid culture under the same treatment conditions to start the next passage.

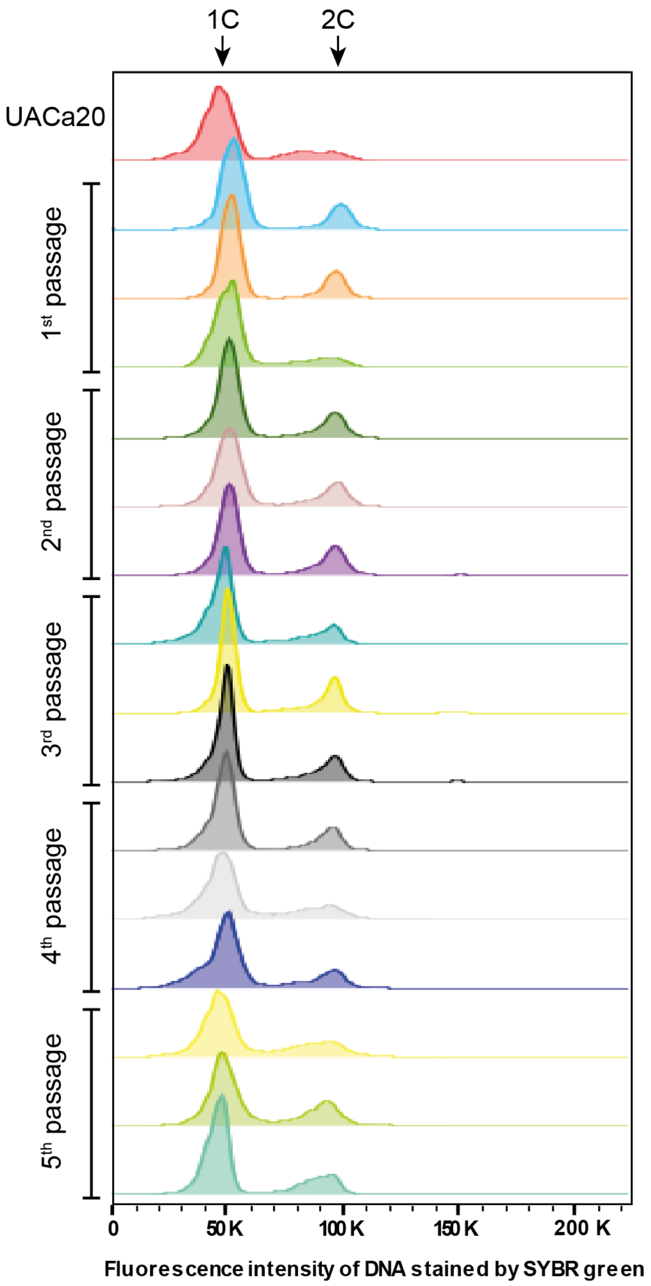


**Figure S2. Cell cycle profile of *Candida auris* UACa20 and of isolates derived by heat stress from it.** Histogram showing cell cycle profiles obtained by flow cytometry after staining DNA with SYBR green of *C. auris* strain UACa20 and its microevolved derivatives (Table S1). The isolates derived by heat stress from *C. auris* UACa20 through 5 consecutive passages have the same cell cycle profile as the parental *C. auris* strain. Approximate position of haploid G1 (1C) and haploid G2 (2C) peaks are indicated at the top.

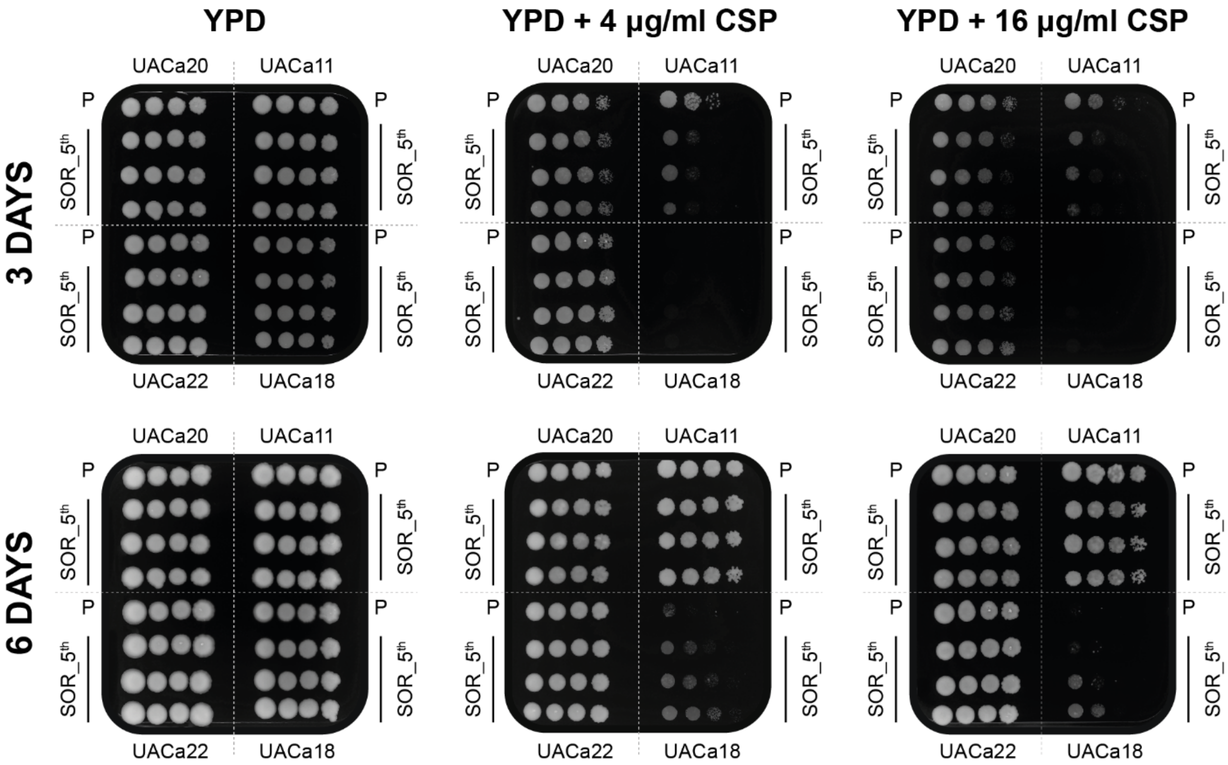


Figure S3. Growth of sorbose-microevolved isolates in presence of caspofungin (CSP). Spot assays on YPD (control), and YPD containing 4 or 16 µg/ml CSP after 3 and 6 days at 30°C of isolates obtained after the 5th passage in the presence of 2% sorbose in synthetic defined medium at 30°C (SOR_5th) from parental strains (P) UACa11, UACa18, UACa20, and UACa22. Serial dilutions contain $10^5$, $10^4$, $10^3$, and $10^2$ cells.

**Table S1.** Details of yeast strains used in this study

| Strain | Collection No. | Species | Relevant genotype/Clade | Site of isolation | Known drug resistances[a] | Origin/Reference |
|---|---|---|---|---|---|---|
| 470026 | UACa1 | *Candida auris* | WT, S. Asia (India) | BSI | FCZ, CSP | A. Chakrabarti[1] |
| 470027 | UACa2 | *Candida auris* | WT, S. Asia (India) | BSI | FCZ, VCZ, CSP | A. Chakrabarti[1] |
| 470028 | UACa3 | *Candida auris* | WT, S. Asia (India) | BSI | FCZ, CSP | A. Chakrabarti[1] |
| 470029 | UACa4 | *Candida auris* | WT, S. Asia (India) | BSI | FCZ, VCZ, CSP | A. Chakrabarti[1] |
| 470030 | UACa5 | *Candida auris* | WT, S. Asia (India) | BSI | FCZ, VCZ, CSP | A. Chakrabarti[1] |
| NCPF8980#9 | UACa6 | *Candida auris* | WT, S. Africa | BSI | FCZ, CSP | E. Johnson |
| NCPF8984#15 | UACa7 | *Candida auris* | WT, E. Asia (Japan) | unknown | FCZ | E. Johnson |
| NCPF8985#20 | UACa8 | *Candida auris* | WT, S. Asia (India) | wound | FCZ, ISA, PSZ, VCZ, 5-FC, AFG | E. Johnson |
| NCPF13001#16 | UACa9 | *Candida auris* | WT, S. Asia (India) | unknown | FCZ | E. Johnson |
| NCPF13005#95 | UACa10 | *Candida auris* | WT, S. Africa | urine | FCZ, VCZ, AFG, AMB, CSP | E. Johnson |
| VPCI479/P/13 | UACa11 | *Candida auris* | WT, S. Asia (India) | BSI | FCZ | A. Chowdhary[2] |
| RBH7723 | UACa12 | *Candida auris* | WT, Royal Brompton hospital | wound | CSP[a] | S. Schelenz[3] |
| RBH7728 | UACa13 | *Candida auris* | WT, Royal Brompton hospital | hospital environment | sensitive | S. Schelenz[3] |
| RBH7745 | UACa14 | *Candida auris* | WT, Royal Brompton hospital | skin swab | FCZ, VCZ, CSP | S. Schelenz[3] |
| RBH7748 | UACa15 | *Candida auris* | WT, Royal Brompton hospital | nose swab | FCZ, VCZ, CSP | S. Schelenz[3] |
| B11220 | UACa18 | *Candida auris* | WT, E. Asia (Japan) | auditory canal | FCZ, VCZ | S. Lockhart[4] |
| B11109 | UACa19 | *Candida auris* | WT, S. Asia (Pakistan) | burn wound | CSP | S. Lockhart[4] |
| B11221 | UACa20 | *Candida auris* | WT, S. Africa | BSI | FCZ, CSP | S. Lockhart[4] |
| B11222 | UACa21 | *Candida auris* | WT, S. Africa | BSI | FCZ, CSP | S. Lockhart[4] |
| B11244 | UACa22 | *Candida auris* | WT, S. America (Venezuela) | BSI | FCZ, VCZ, CSP | S. Lockhart[4] |
| B11245 | UACa23 | *Candida auris* | WT, S. America (Venezuela) | BSI | FCZ, VCZ, CSP | S. Lockhart[4] |
| B8441 | UACa24 | *Candida auris* | WT, S. Asia (Pakistan) | BSI | CSP | S. Lockhart[4] |
| B11098 | UACa25 | *Candida auris* | WT, S. Asia (Pakistan) | BSI | FCZ | S. Lockhart[4] |
| B11203 | UACa26 | *Candida auris* | WT, S. Asia (India) | BAL | FCZ, 5-FC | S. Lockhart[4] |
| B11205 | UACa27 | *Candida auris* | WT, S. Asia (India) | chest wound | FCZ, VCZ, 5-FC | S. Lockhart[4] |
| CBS10913T | UACa83 | *Candida auris* | WT, E. Asia (Japan) | auditory canal | none | CBS-KNAW collection[5] |

| | | | | | | |
|---|---|---|---|---|---|---|
| BY4741 | UACa35 | *Saccharomyces cerevisiae* | *MAT***a** *his3Δ1 leu2Δ0 met15Δ0 ura3Δ0* | n/a | n/a | lab strain[6] |
| BY4742 | UACa36 | *Saccharomyces cerevisiae* | *MATα his3Δ1 leu2Δ0 lys2Δ0 ura3Δ0* | n/a | n/a | lab strain[6] |
| BY4743 | UACa37 | *Saccharomyces cerevisiae* | *MAT***a***/α his3Δ1/his3Δ1 leu2Δ0/leu2Δ0 LYS2/lys2Δ0 MET15/met15Δ0* ura3Δ0/*ura3Δ0* | n/a | n/a | lab strain; cross of BY4741×BY4742 |
| SC5314 | UACa38 | *Candida albicans* | WT | n/a | n/a | lab strain[7] |

[a]Using EUCAST clinical breakpoints for *Candida albicans*: (http://www.eucast.org/fileadmin/src/media/PDFs/EUCAST_files/AFST/Clinical_breakpoints/Antifungal_breakpoints_v_9.0_180212.pdf)

Abbreviations: BAL = broncho-alveolar lavage, BSI = bloodstream infection, FCZ = fluconazole, ISA = Isavuconazole, PSZ = Posaconazole, VCZ = Voriconazole, CSP = Caspofungin, AFG = Anidulafungin, 5-FC = flucytosine, AMB = Amphotericin B.

## Supplementary References


1. Chakrabarti A, Sood P, Rudramurthy SM, et al. Incidence, characteristics and outcome of ICU-acquired candidemia in India. *Intensive Care Med*. 2014;41(2):285-295. doi:10.1007/s00134-014-3603-2.
2. Sharma C, Kumar N, Meis JF, Pandey R, Chowdhary A. Draft genome sequence of a FCZ-resistant *Candida auris* strain from a candidemia patient in India. *Genome Announc*. 2015;3(4):e00722-15. doi:10.1128/genomeA.00722-15.
3. Schelenz S, Hagen F, Rhodes JL, et al. First hospital outbreak of the globally emerging *Candida auris* in a European hospital. *Antimicrob Resist Infect Control*. 2016;5(1):35. doi:10.1186/s13756-016-0132-5.
4. Lockhart SR, Etienne KA, Vallabhaneni S, et al. Simultaneous emergence of multidrug-resistant *Candida auris* on 3 continents confirmed by whole-genome sequencing and epidemiological analyses. *Clin Infect Dis*. 2017;64(2):134-140. doi:10.1093/cid/ciw691.
5. Satoh K, Makimura K, Hasumi Y, Nishiyama Y, Uchida K, Yamaguchi H. *Candida auris* sp. nov., a novel ascomycetous yeast isolated from the external ear canal of an inpatient in a Japanese hospital. *Microbiol Immunol*. 2009;53(1):41-44. doi:10.1111/j.1348-0421.2008.00083.x.
6. Brachmann CB, Davies A, Cost GJ, et al. Designer deletion strains derived from *Saccharomyces cerevisiae* S288C: a useful set of strains and plasmids for PCR-mediated gene disruption and other applications. *Yeast*. 1998;14(2):115-132. doi:10.1002/(SICI)1097-0061(19980130)14:2<115::AID-YEA204>3.0.CO;2-2.
7. Odds FC, Brown AJP, Gow NAR. *Candida albicans* genome sequence: a platform for genomics in the absence of genetics. *Genome Biol*. 2004;5(7):230. doi:10.1186/gb-2004-5-7-230.
